# Comparative transcriptomic and proteomic analysis of *Arthrobacter* sp. CGMCC 3584 responding to dissolved oxygen for cAMP production

**DOI:** 10.1038/s41598-017-18889-4

**Published:** 2018-01-19

**Authors:** Huanqing Niu, Junzhi Wang, Wei Zhuang, Dong Liu, Yong Chen, Chenjie Zhu, Hanjie Ying

**Affiliations:** 1 State Key Laboratory of Materials-Oriented Chemical Engineering, College of Biotechnology and Pharmaceutical Engineering, Nanjing Tech University, No.30, Puzhu South Road, Nanjing, 211816 China; 2 National Engineering Technique Research Center for Biotechnology, No.30, Puzhu South Road, Nanjing, 211816 China; 3 Jiangsu National Synergetic Innovation Center for Advanced Materials (SICAM), No.30, Xinmofan Road, Nanjing, 210009 China

## Abstract

*Arthrobacter* sp. CGMCC 3584 is able to produce high yields of extracellular cyclic adenosine monophosphate (cAMP), which plays a vital role in the field of treatment of disease and animal food, during aerobic fermentation. However, the molecular basis of cAMP production in *Arthrobacter* species is rarely explored. Here, for the first time, we report the comparative transcriptomic and proteomic study of *Arthrobacter* cells to elucidate the higher productivity of cAMP under high oxygen supply. We finally obtained 14.1% and 19.3% of the *Arthrobacter* genome genes which were up-regulated and down-regulated notably, respectively, with high oxygen supply, and identified 54 differently expressed proteins. Our results revealed that high oxygen supply had two major effects on metabolism: inhibition of glycolysis, pyruvate metabolism, nitrogen metabolism, and amino acid metabolism (histidine, branched-chain amino acids and glutamate metabolism); enhancement of the tricarboxylic acid cycle and purine metabolism. We also found that regulation of adenylate cyclase and phosphodiesterase was not significant under high oxygen supply, suggesting efficient cAMP export might be important in cAMP production. These findings may contribute to further understanding of capacities of *Arthrobacter* species and would be highly useful in genetic regulation for desirable production.

## Introduction

Cyclic adenosine monophosphate (cAMP) is an important compound which exists in many living cells, and participates in the regulation of physiological actions, such as cell proliferation and differentiation, hormones synthesis and secretion, membrane protein activity, nervous activity, gene expression and so on^1–3^. Based on its functions, cAMP is used in pharmaceuticals and as feed additives. The industrial production of cAMP through microorganism attracts attention of researchers for low cost and environmental friendliness. The microbes, including *Microbacterium*, *Arthrobacter*, *Brevibacterium liquefacien*, *Corynebacterium roseoparaffineus*, *Corynebacterium murisepticum*, were reported to be able to produce cAMP^4,5^.

Our previous studies have demonstrated that a strain, *Arthrobacter* sp. CGMCC 3584, was capable of producing high yields of cAMP in culture media, through optimization of culture conditions and metabolic regulation on the fermentation process^6–8^. *Arthrobacter* species are among the most common aerobic culturable bacteria and exist in the nature widely, mainly in soil and in some extreme environments^9,10^. Their remarkable tolerance to various stresses contributes to the application of *Arthrobacter* species in contaminant degradation in complex and volatile environments^11^. Most genome and transcriptome researches of this genus focus on the genetic basis of the biodegradation and survival capacities, and available microarray data are rarely thus far. In the present study, DNA microarray-based transcriptomic analysis and two-dimensional (2D) gel electrophoresis technology and mass spectrum (MS) based proteomic analysis of *Arthrobacter* cells cultured under various oxygen supply conditions was conducted to dissect the molecular regulatory mechanism of cAMP production.

It has been reported that oxygen supply, which could influence nutrients uptake, cell metabolism and product yield, plays an important role in aerobic fermentation^12^. Oxygen can affect the expression patterns of genes involved in various cell functions including carbon metabolism, iron uptake and stress response^13^. Many transcriptomic studies were conducted to gain insight into the mechanism of fermentative lifestyle evolution under different conditions of oxygen availability. For instance, a microarray study on *Kluyveromyces lactis* showed that the availability of oxygen determined the fermentation pattern, with increasing the glucose metabolism and reducing fluxes in the pentose phosphate pathway under oxygen-limited conditions^14^. Another DNA microarray analysis of the full transcriptome of the *Bordetella pertussis* bacterium revealed that oxygen limitation during cultivation had a fully reversible effect on gene expression. Microarray analysis was also used to investigate the global gene expression of *Lactococcus lactis* subsp. *lactis* and results indicated that trehalose and GTP were implicated in bacterial adaptation to oxidative stress^15^. Our previous experiments confirmed dissolved oxygen (DO) level definitely affected cAMP production during fermentation^16^. However, the intrinsic relationship between oxygen supply and cAMP biosynthesis is still not so clear. Thus far, no transcriptomic and proteomic analysis of cAMP-producing bacterial cells has been reported. In this work, we try to comprehensively explore the underlying mechanisms of oxygen supply on cAMP biosynthesis, which will help to better understand the metabolic regulation mechanism of *Arthrobacter* species and provide valuable information for further improvement of cAMP production.

## Results

### Oxygen supply and cAMP production

**Figure 1:**
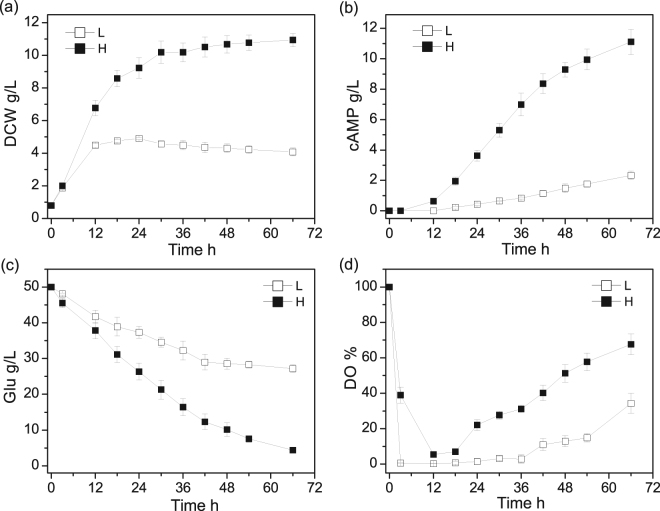
The time courses of DCW (**a**) and cAMP (**b**), and DO (**c**) and glucose (**d**) in the 5-L bioreactor fermentation with low oxygen supply (L) and high oxygen supply (H).

In the 5-L fermenters, two batches of cAMP fermentation were carried out with agitation set at 150 and 350 r/min to construct low oxygen supply and high oxygen supply conditions, respectively. As shown in Fig. 1, the DO level showed a sharp drop in the initial phase of fermentation both in low and high oxygen supply. The DO concentration reduced to nearly zero at 3 h and began to rise until 36 h with 150 r/min agitation, while the DO was at a much higher level with 350 r/min agitation. After 66 h fermentation, the consumption of glucose under high oxygen supply was almost twice as under low oxygen supply. The highest DCW values and cAMP yields reached 4.91 ± 0.05 g/L and 2.34 ± 0.21 g/L, respectively, under low oxygen supply, and 10.94 ± 0.41 g/L and 11.17 ± 0.81 g/L, respectively, under high oxygen supply. The final cAMP yield on DCW reached 0.57 ± 0.027 g/g DCW and 1.02 ± 0.03 g/g DCW under low and high oxygen supply respectively. This result showed that the oxygen supply condition had a greater influence on cAMP production than cell growth.

### General changes on transcriptional level

**Figure 2:**
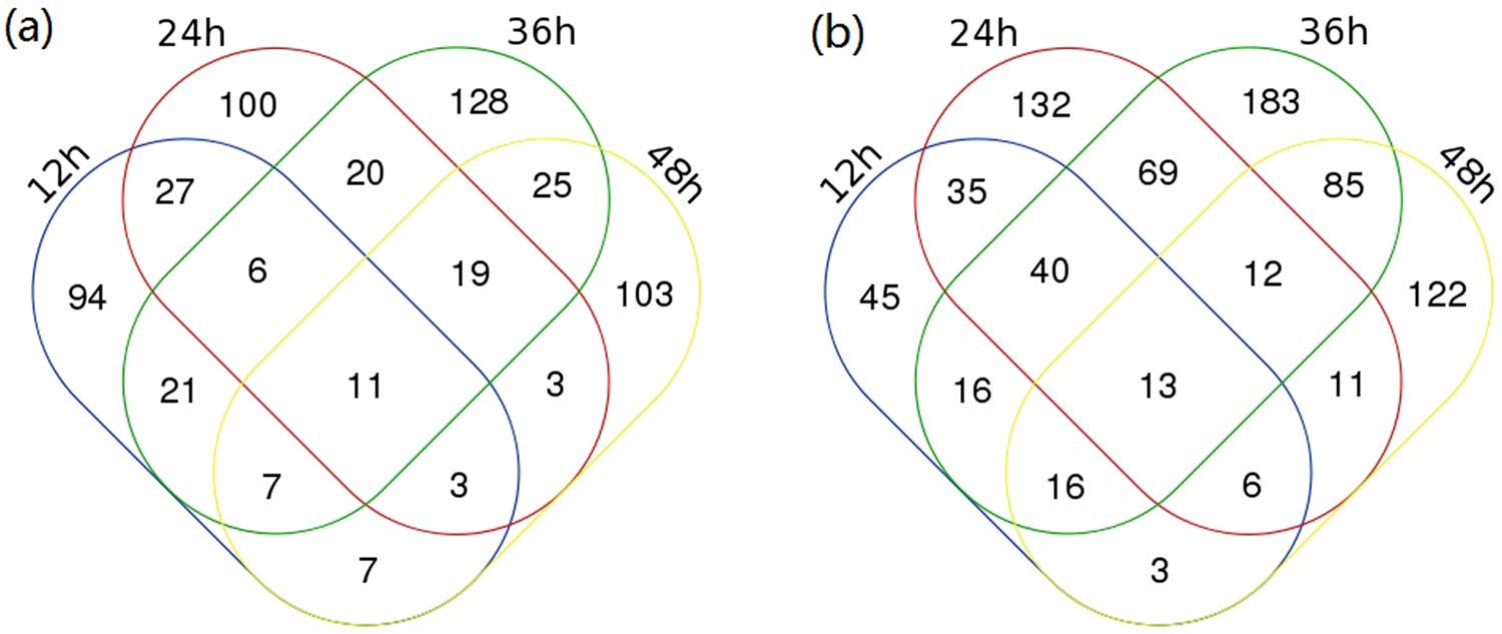
Venn diagram of the up-regulated (**a**) or down-regulated (**b**) genes (more than twofold) with high oxygen supply of 12 h, 24 h, 36 h and 48 h samples.

Cell samples of *Arthrobacter* sp. CGMCC 3584 were collected after 12 h, 24 h, 36 h and 48 h fermentation under different oxygen supply conditions for microarray analysis. The oxygen supply condition appeared to have a significant effect on global transcript levels in *Arthrobacter* sp. CGMCC 3584 for cAMP fermentation. As shown in Supplementary Table S1, 350, 507, 671 and 446 genes were identified to differentially express using an empirical 2-fold criterion, for 12 h, 24 h, 36 h and 48 h samples, respectively. Among these differentially expressed genes, 176, 189, 237 and 178 genes were up-regulated (ratio_high/low_ ≥ 2) with high oxygen supply for 12 h, 24 h, 36 h and 48 h samples, respectively. While compared to the low oxygen supply condition, 174, 318, 434 and 268 genes were down-regulated (ratio_high/low _ ≤ 0.5) at 12 h, 24 h, 36 h and 48 h respectively. As shown in Fig. 2, a total of 574 genes, representing 14.1% of the *Arthrobacter* genome genes, exhibited up-regulation of mRNA expression level and 788 genes, representing 19.3% of the *Arthrobacter* genome genes, were down-regulated significantly with high oxygen supply. Regarding the differentially expressed genes shared by the samples of four time points, 11 were increased in relative expression level (Fig. 2a) and 13 were decreased in relative expression level (Fig. 2b).

**Figure 3:**
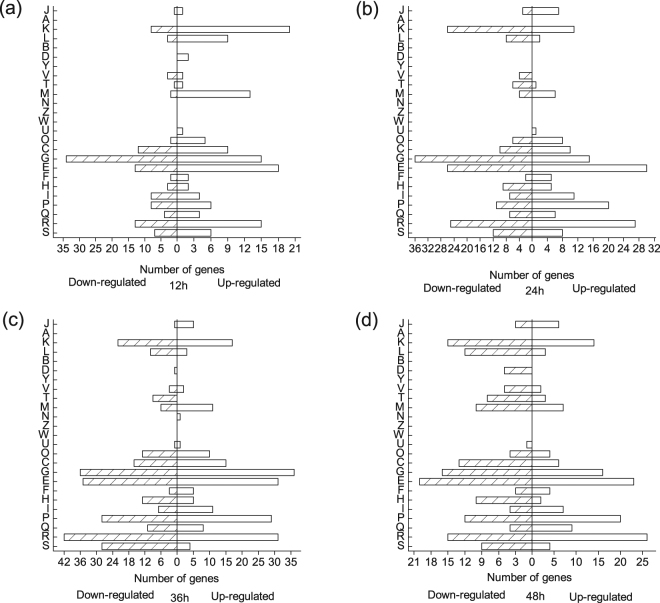
Function classifications of differentially expressed genes (fold changes of at least 2) at 12 h (**a**), 24 h (**b**), 36 h (**c**) and 48 h (**d**), respectively. Abbreviations: J, Translation, ribosomal structure and biogenesis; A, RNA processing and modification; K, Transcription; L, Replication, recombination and repair; B, Chromatin structure and dynamics; D, Cell cycle control, cell division, chromosome partitioning; Y, Nuclear structure; V, Defense mechanisms; T, Signal transduction mechanisms; M, Cell wall/membrane/envelope biogenesis; N, Cell motility; Z, Cytoskeleton; W, Extracellular structures; U, Intracellular trafficking, secretion, and vesicular transport; O, Posttranslational modification, protein turnover, chaperones; C, Energy production and conversion; G, Carbohydrate transport and metabolism; E, Amino acid transport and metabolism; F, Nucleotide transport and metabolism; H, Coenzyme transport and metabolism; I, Lipid transport and metabolism; P, Inorganic ion transport and metabolism; Q, Secondary metabolites biosynthesis, transport and catabolism; R, General function prediction only; S, Function unknown.

These differentially expressed genes fell into major Clusters of Orthologous Groups (COG) categories (shown in Fig. 3, based on the NCBI-COG database). The largest group with altered transcriptional levels belonged to the group involved in carbohydrate transport and metabolism (11.9% up-regulated and 15.6% down-regulated). Other large functional groups of differentially expressed genes changed significantly included amino acid transport and metabolism (14.8% up-regulated and 12.1% down-regulated), inorganic ion transport and metabolism (10.9% up-regulated and 7.6% down-regulated), and transcription(9.0% up-regulated and 9.1% down-regulated). And 5.3% genes were annotated as function unknown. The genes associated with RNA processing and modification, chromatin structure and dynamics, nuclear structure, cytoskeleton and extracellular structures did not show any significant changes with high oxygen supply at all four time points.

### General changes on proteomic level

**Figure 4:**
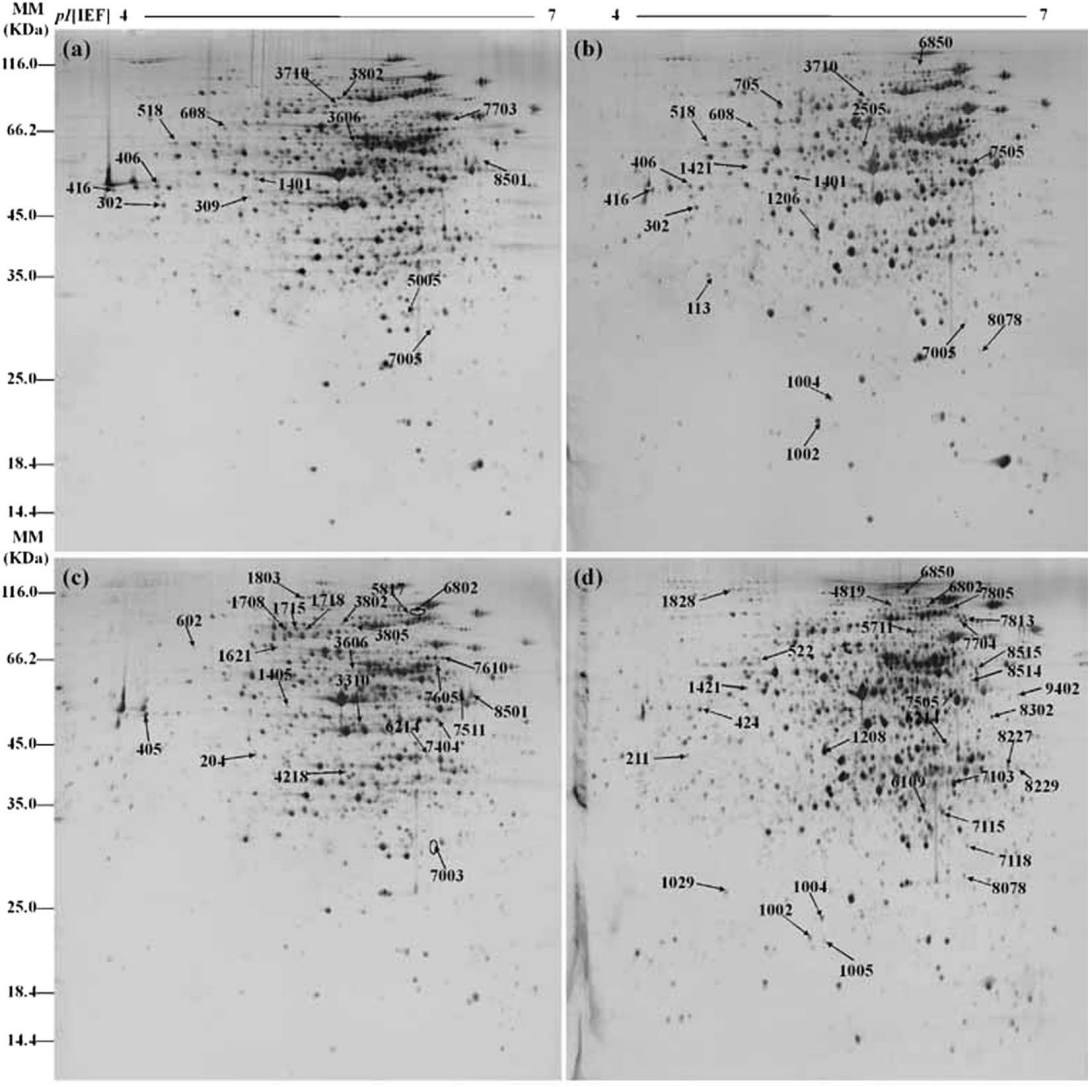
2D gel maps of proteins extracted from *Arthrobacter* sp. CGMCC 3584 at 12 h with low oxygen supply (**a**), at 12 h with high oxygen supply (**b**), at 36 h with low oxygen supply (**c**) and at 36 h with high oxygen supply (**d**). Differentially expressed protein spots which were identified successfully for each treatment are marked with numbers.

Cell samples of *Arthrobacter* sp. CGMCC 3584 were collected after 12 h and 36 h fermentation under different oxygen supply conditions for 2D gel separation. The 2D gel images were shown in Fig. 4. Protein expression level ratios (high oxygen supply/low oxygen supply) ≥2.5 or ≤0.4 (p < 0.05) were considered to be significantly regulated. As a result, a total of 67 differentially expressed protein spots showed changes of significance in their intensities under different oxygen supply (listed in Supplementary Table S2). For 12 h samples, 11 protein spots were significantly up-regulated with high oxygen supply (ratio_high/low_ ≥ 2.5), whereas 5 spots were down-regulated (ratio_high/low_ ≤ 0.4). For 36 h samples, 38 protein spots were significantly up-regulated with high oxygen supply (ratio_high/low_ ≥ 2.5), whereas 22 spots were down-regulated (ratio_high/low_ ≤ 0.4). These protein spots were further analyzed by MALDI-TOF/TOF-MS and 54 spots among them were successfully identified (listed in Table 1). Among these 54 spots, twelve different protein spots were identified to represent six gene products, including succinyl-CoA synthetase subunit beta (ID: 8229, 8515), molecular chaperone DnaK (ID: 1005, 1029), elongation factor Ts (ID: 1208, 4218), threonyl-tRNA synthetase (ID: 4819, 3802, elongation factor Tu (ID: 8227, 8501), and phosphomannomutase (ID: 7610, 8078). So we finally obtained 48 different proteins.

**Table 1:** Identified differentially expressed proteins responding to different oxygen supply in *Arthrobacter* sp. CGMCC 3584.

Spot no.	NCBI accession no.	Protein name	Species	Proteome fold changes				Experimental mass (kDa) and pI	Score
				12 h	p-value	36 h	p-value		
113	gi|72163016	50 S ribosomal protein L17	*Thermobifida fusca* YX	2.78	5.57E-06			43.78/4.82	58
204	gi|50955503	succinyl-CoA synthetase subunit alpha	*Leifsonia xyli* subsp. *xyli* str. CTCB07			0.38	1.71E-02	52.87/5.29	68
211	gi|220912059	ribose-phosphate pyrophosphokinase	*Arthrobacter chlorophenolicus* A6			3.79	2.93E-04	52.19/4.7	74
405	gi|325962845	protein RecA	*Arthrobacter phenanthrenivorans* Sphe3			0.26	1.35E-03	64.93/4.58	508
424	gi|444307928	threonine synthase	*Arthrobacter* sp. SJCon			2.57	4.30E-04	63.44/4.76	154
522	gi|325962559	serine hydroxymethyltransferase	*Arthrobacter phenanthrenivorans* Sphe3			3.38	1.88E-07	76.21/5.18	247
602	gi|119961574	6-phosphogluconate dehydrogenase	*Arthrobacter* aurescens TC1			0.34	7.31E-04	83.35/4.99	186
705	gi|220912286	CTP synthetase	*Arthrobacter chlorophenolicus* A6	2.99	6.63E-07			90.53/5.3	399
1002	gi|116670901	ribonucleotide-diphosphate reductase subunit beta	*Arthrobacter* sp. FB24	3.94	1.22E-02	2.55	1.48E-02	23.42/5.49	58
1004	gi|220910796	peptidyl-prolyl isomerase	*Arthrobacter chlorophenolicus* A6	4.79	1.87E-03	21.88	1.75E-06	25.6/5.56	252
1005	gi|3023655	Chaperone protein DnaK				3.94	2.02E-03	22.84/5.59	71
1029	gi|116672350	molecular chaperone DnaK	*Arthrobacter* sp. FB24			11.27	4.72E-04	28.73/4.97	107
1206	gi|220913188	winged helix family two component transcriptional regulator	*Arthrobacter chlorophenolicus* A6	3.59	3.09E-04			52.01/5.5	259
1208	gi|220912155	elongation factor Ts	*Arthrobacter chlorophenolicus* A6			4.37	1.18E-04	53.72/5.59	80
1405	gi|325963192	triosephosphate isomerase	*Arthrobacter phenanthrenivorans* Sphe3			0.07	4.37E-03	66.41/5.5	81
1421	gi|119963487	sugar ABC transportor, ATP-binding protein	*Arthrobacter aurescens* TC1	3.12	2.01E-05	5.50	6.09E-04	69.45/5.09	107
1708	gi|116669060	NAD synthetase	*Arthrobacter* sp. FB24			0.22	5.15E-03	93.34/5.48	146
1715	gi|119950507	dihydroxy-acid dehydratase	*Arthrobacter aurescens* TC1			0.29	9.31E-03	93.26/5.55	112
1718	gi|119964210	aspartyl-tRNA synthetase	*Arthrobacter aurescens* TC1			0.29	4.78E-04	93.61/5.6	64
1803	gi|359777979	catalase	*Arthrobacter globiformis* NBRC 12137			0.36	1.40E-02	107.08/5.58	91
1828	gi|220911112	ATPase AAA	*Arthrobacter chlorophenolicus* A6	2.35	4.62E-02	6.22	4.39E-05	106.67/4.98	495
2505	gi|325963975	glutamate-1-semialdehyde 2,1-aminomutase	*Arthrobacter phenanthrenivorans* Sphe3	2.80	6.70E-03			75.36/5.77	241
3310	gi|116670653	FeS assembly ATPase SufC	*Arthrobacter* sp. FB24			0.07	2.43E-05	62.6/5.94	172
3606	gi|119964256	glycogen synthase	*Arthrobacter aurescens* TC1	0.24	8.44E-05	0.25	2.17E-06	79.21/5.89	239
3802	gi|116670864	threonyl-tRNA synthetase	*Arthrobacter* sp. FB24	0.28	1.24E-03	0.31	8.69E-03	96.06/5.83	182
3805	gi|220912455	pyruvate kinase	*Arthrobacter chlorophenolicus* A6			0.33	3.49E-03	95.81/5.89	118
4218	gi|220912155	elongation factor Ts	*Arthrobacter chlorophenolicus* A6			0.35	6.53E-05	51.32/5.87	219
4819	gi|220912797	threonyl-tRNA synthetase	*Arthrobacter chlorophenolicus* A6			3.29	4.54E-05	101.65/6.01	189
5005	gi|325962142	uracil phosphoribosyltransferase	*Arthrobacter phenanthrenivorans* Sphe3	0.36	9.82E-05			38.3/6.2	294
5711	gi|325964075	chaperonin GroL	*Arthrobacter phenanthrenivorans* Sphe3			3.56	1.20E-04	90.28/6.15	713
6109	gi|220912628	short chain dehydrogenase	*Arthrobacter chlorophenolicus* A6			15.97	5.33E-06	41.96/6.21	329
6850	gi|325963015	aconitase	*Arthrobacter phenanthrenivorans* Sphe3	2.77	1.46E-06	7.52	2.88E-10	106.48/6.08	723
7003	gi|325963765	ketol-acid reductoisomerase	*Arthrobacter phenanthrenivorans* Sphe3			0.21	5.89E-07	37.9/6.38	413
7103	gi|116669084	fructose-bisphosphate aldolase	*Arthrobacter* sp. FB24			3.72	2.21E-05	47.01/6.39	305
7115	gi|325964935	phosphoribosylanthranilate isomerase	*Arthrobacter phenanthrenivorans* Sphe3			17.86	9.03E-08	41.64/6.32	160
7118	gi|325963063	response regulator with antiterminator output domain	*Arthrobacter phenanthrenivorans* Sphe3			7.38	1.21E-04	35.6/6.47	93
7210	gi|116668714	inorganic diphosphatase	*Arthrobacter* sp. FB24			2.50	6.31E-03	49.57/6.46	109
7404	gi|220913400	DNA-directed RNA polymerase subunit alpha	*Arthrobacter chlorophenolicus* A6			0.29	7.77E-03	64.16/6.39	400
7505	gi|325962588	enolase	*Arthrobacter phenanthrenivorans* Sphe3	2.60	8.29E-05	9.28	1.76E-02	70.14/6.4	760
7511	gi|325963191	phosphoglycerate kinase	*Arthrobacter phenanthrenivorans* Sphe3			0.30	9.03E-04	72.17/6.53	320
7605	gi|325962719	aspartyl/glutamyl-tRNA amidotransferase subunit A	*Arthrobacter phenanthrenivorans* Sphe3			0.37	1.20E-02	81.67/6.38	623
7610	gi|220911593	phosphomannomutase	*Arthrobacter chlorophenolicus* A6			0.32	3.72E-03	83.91/6.42	291
7703	gi|116672548	phosphoenolpyruvate–protein phosphotransferase	*Arthrobacter* sp. FB24	0.23	2.60E-02			87.25/6.42	219
7704	gi|220914230	glucose-methanol-choline oxidoreductase	*Arthrobacter chlorophenolicus* A6			3.77	1.62E-04	95.29/6.43	80
7805	gi|119961649	2-oxoglutarate dehydrogenase, E2 component, dihydrolipoamide succinyltransferase	*Arthrobacter aurescens* TC1			3.11	3.31E-05	100.9/6.37	445
7813	gi|116670612	30 S ribosomal protein S1	*Arthrobacter* sp. FB24			4.09	3.64E-04	95.41/6.46	434
8078	gi|220911593	phosphomannomutase	*Arthrobacter chlorophenolicus* A6	5.20	1.19E-03	9.14	1.56E-05	31.22/6.45	606
8227	gi|116671525	elongation factor Tu	*Arthrobacter* sp. FB24			6.29	2.15E-03	50.02/6.72	142
8229	gi|116669380	succinyl-CoA synthetase subunit beta	*Arthrobacter* sp. FB24			6.56	1.62E-04	49.57/6.78	227
8302	gi|220911115	dihydroxyacetone kinase subunit DhaK	*Arthrobacter chlorophenolicus* A6			4.18	1.09E-03	62.6/6.61	662
8501	gi|116671525	elongation factor Tu	*Arthrobacter* sp. FB24	0.10	4.81E-06	0.04	1.23E-04	72.51/6.58	224
8514	gi|444304706	F0F1 ATP synthase subunit beta	*Arthrobacter* sp. SJCon			6.67	6.23E-05	72.83/6.49	461
8515	gi|116669380	succinyl-CoA synthetase subunit beta	*Arthrobacter* sp. FB24			10.20	1.84E-04	76.21/6.52	292
9402	gi|116670134	cell division protein FtsZ	*Arthrobacter* sp. FB24			5.01	5.66E-05	68.1/6.79	78

**Figure 5:**
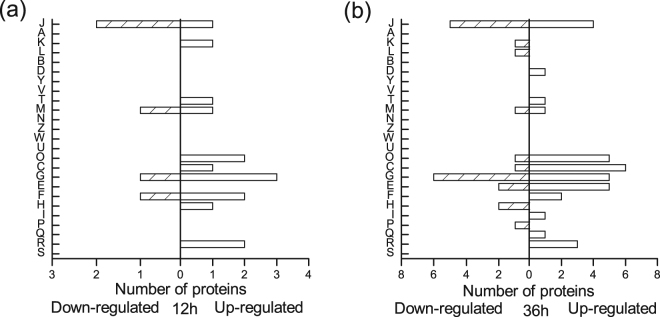
Function classifications of differentially expressed proteins (fold changes of at least 2.5) at 12 h (**a**) and 36 h (**b**). For abbreviations, see the legend to Fig. 3.

The distribution of the differentially expressed proteins over the different COG categories was exhibited in Fig. 5. Among these successfully identified spots, 32, 11 and 10 proteins belonged to metabolism, information storage and processing, and cellular processes and signaling, respectively. And 3 proteins were poorly characterized. According to the COG categories, the up-regulated proteins under high oxygen supply were mainly involved in carbohydrate transport and metabolism, energy production and conversion, and posttranslational modification, protein turnover, chaperones. The majority of the down-regulated proteins were classified as carbohydrate transport and metabolism, and translation, ribosomal structure and biogenesis.

### Quantitative real-time PCR (qRT-PCR) validation of the genes transcription levels

In order to validate the microarray data, we used qRT-PCR to further investigate the mRNA expression of five selected genes for 12 h and 36 h samples, including ribose-phosphate pyrophosphokinase (gismo_orf1245), pyruvate kinase (gismo_orf2621), fructose-bisphosphate aldolase (gismo_orf2812), succinyl-CoA synthetase subunit beta (gismo_orf3726) and phosphoglycerate kinase (gismo_orf3896). As shown in Table 2, the mRNA ratios obtained by qRT-PCR were mostly in good agreement with the fold changes from the microarray data analysis, except the expressions of fructose-bisphosphate aldolase (12 h samples) and phosphoglycerate kinase (36 h samples) showed some inconsistency between qRT-PCR and microarray results. In addition, the relevant protein fold changes were also shown in Table 2. The protein abundance of ribose-phosphate pyrophosphokinase (spot 211), pyruvate kinase (spot 3805), fructose-bisphosphate aldolase (spot 7103) and succinyl-CoA synthetase subunit beta (spot 8229 and 8515) exhibited the similar fold changes with the relevant qRT-PCR results at 12 h or 36 h with high oxygen supply. And the results of qRT-PCR and protein analysis of phosphoglycerate kinase (spot 7511) were inconsistent for both 12 h and 36 h samples.

**Table 2:** Correlation between microarray and qRT-PCR results.

Gene_ID	Protein spot no.	Gene description	Microarray fold change		Protein fold change		qRT-PCR fold chang	
			12 h	36 h	12 h	36 h	12 h	36 h
gismo_orf1245	211	ribose-phosphate pyrophosphokinase	1.69	2.09	0.97	3.79	2.48	3.48
gismo_orf2621	3805	pyruvate kinase	0.44	0.52	0.49	0.33	0.56	0.60
gismo_orf2812	7103	fructose-bisphosphate aldolase	0.94	0.66	1.23	3.72	1.38	0.92
gismo_orf3726	8229 and 8515	succinyl-CoA synthetase subunit beta	1.19	2.90	2.33 and 1.03	10.20 and 6.56	1.35	4.82
gismo_orf3896	7511	phosphoglycerate kinase	0.43	0.40	1.09	0.30	0.49	1.01

## Discussion

**Figure 6:**
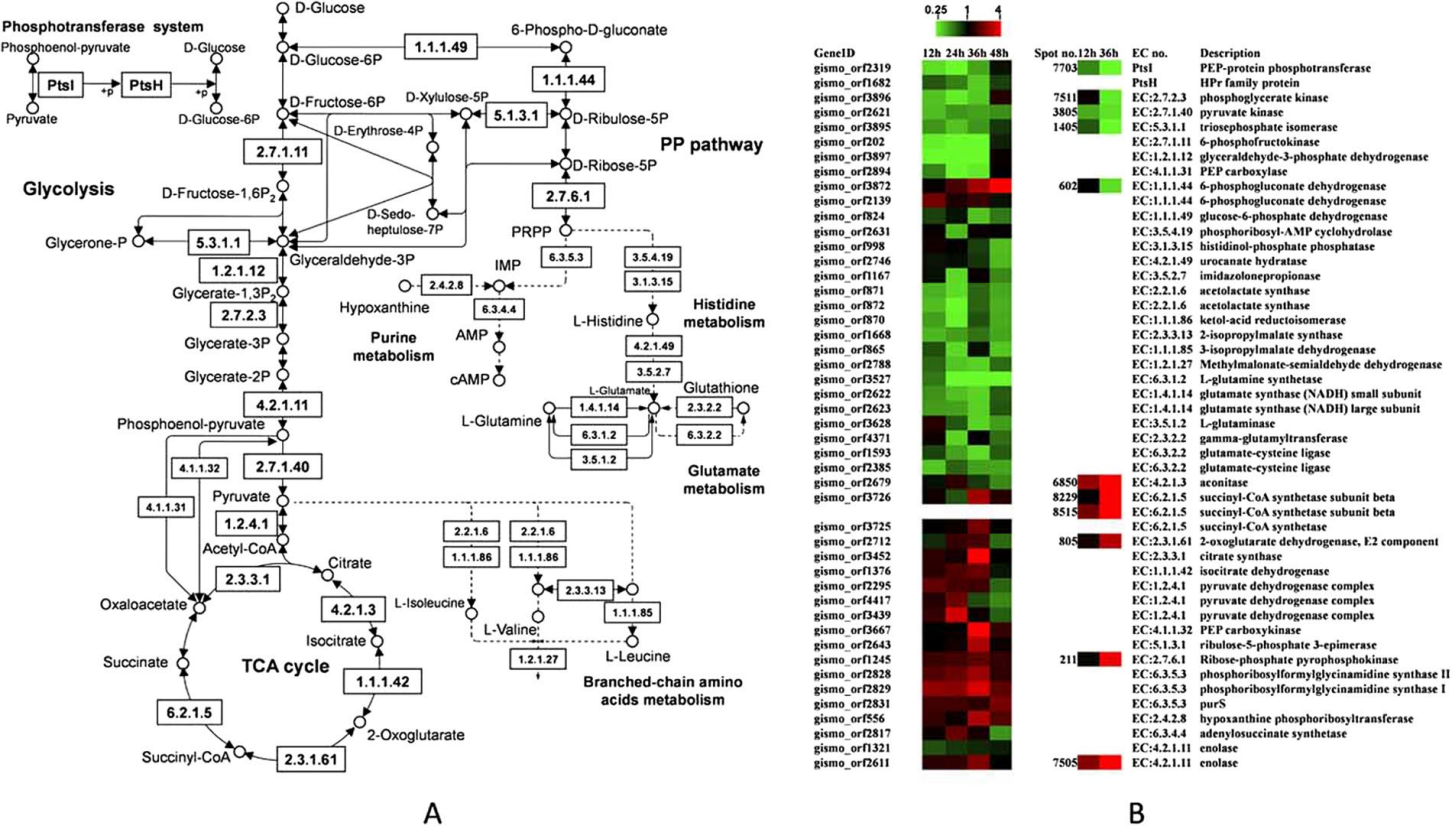
The central carbon metabolic network of *Arthrobacter* sp. CGMCC 3584 (**A**) and 2-fold changes in expression levels of the related genes and proteins responding to different oxygen supply (**B**). The pathway is based on the KEGG pathway database (http://www.genome.jp/kegg/pathway.html)^55^.

Limited information is available on the molecular mechanism of cAMP-producing fermentation. Here, we reported a comprehensive transcriptome and proteome study to characterize the gene and protein expression profiles of a cAMP-producing strain, *Arthrobacter* sp. CGMCC 3584, responding to DO level. The important genes and proteins with significantly differential expressions, related to the central carbon metabolic network, were shown in Fig. 6.

The phosphoenolpyruvate (PEP)-dependent phosphotransferase system (PTS) is a major mechanism for uptake of carbohydrates in bacteria, where PEP is used as an energy source and phosphoryl donor. The PTS carries out its catalytic function in sugar transport and phosphorylation by two general components, including enzyme I (EI) and histidine phosphocarrier protein (HPr), and of membrane-bound sugar specific permeases (enzymes II). It is observed that the PTS can regulate the cAMP concentration and adenylate cyclase activity. For example, the addition of glucose and other PTS carbohydrates inhibits cAMP synthesis in *E*. *coli*^17^. Furthermore, the PTS not only controls carbohydrate uptake and metabolism but also influences the utilization of nitrogen and phosphorus. In our study, some proteins involved in glucose utilization were down-regulated under high oxygen supply. One carbohydrate uptake protein, PEP-protein phosphotransferase (spot 7703), exhibited a significant reduction at 12 h as well as its mRNA level (gismo_orf2319) with high oxygen supply. Meanwhile, microarray results indicated that genes encoding HPr family protein (gismo_orf1682), maltose/glucose porter, IIABC component (gismo_orf1998), beta-glucoside-specific, IIABC component (gismo_orf502), and mannitol-specific IIC component (gismo_orf2322) which involved in PTS system were down-regulated significantly.

Three proteins involved in glycolysis, including phosphoglycerate kinase (spot 7511), pyruvate kinase (spot 3805), and triosephosphate isomerase (spot 1405) were also down-regulated significantly with high oxygen supply at 12 h or 36 h, and their transcriptional levels were decreased significantly as well (gismo_orf3896, gismo_orf2621 and gismo_orf3895, respectively). The microarray results also showed that gene expression levels of 6-phospho-beta-glucosidase (gismo_orf501), 6-phosphofructokinase (gismo_orf202) and glyceraldehyde-3-phosphate dehydrogenase (gismo_orf3897) which involved in glycolysis were reduced significantly under high oxygen supply. In addition, the pathways of pyruvate metabolism tended to be restrained under high oxygen supply. The genes encoding PEP carboxylase (gismo_orf2894), 2-isopropylmalate synthase (gismo_orf1668), acetyl-CoA acetyltransferase (gismo_orf220), and ring-cleavage extradiol dioxygenase (gismo_orf817) showed significantly negative changes in their expression levels, except the gene expression level of acetyl/propionyl-CoA carboxylase subunit alpha (gismo_orf1209) was over expressed at 24 h. Under high DO level, the 6-phosphogluconate dehydrogenase (6PGDH, spot 602) which catalyzed the formation of ribulose 5-phosphate from 6-Phospho-D-gluconate, decreased in its abundance obviously at 36 h. Meanwhile, the gene encoding the glucose-6-phosphate dehydrogenase (G6PDH, gismo_orf824) that catalyzed glucose 6-phosphate to 6-phospho-D-glucono-1,5-lactone was also detected to be down-regulated at 36 h. G6PDH and 6PGDH are the key enzymes of pentose phosphate (PP) pathway involving in NADPH production^18^. PP pathway supplies the cell with precursors for the biosynthesis of nucleic acids, amino acids, cofactors and cell wall constituents. These expression profiles mentioned above suggest that high oxygen supply might repress carbohydrate uptake to some extent in this strain. Previous studies also revealed that low oxygen supply promoted glucose metabolism in adenosine fermentation of *Bacillus subtilis*^19^, and low agitation enhanced glucose consumption rate in pyruvate fermentation of *Torulopsis glabrata*^20^.

On the other hand, the proteomic results showed that three TCA cycle-related proteins, aconitase (spot 6850), succinyl-CoA synthetase subunit beta (spot 8229 and 8515), and 2-oxoglutarate dehydrogenase, E2 component, dihydrolipoamide succinyltransferase (spot 7805), were significantly increased in their abundances at 12 h or 36 h with high oxygen supply. Meanwhile, the transcriptome data indicated that genes encoding citrate synthase (gismo_orf3452) and isocitrate dehydrogenase (gismo_orf1376) which were important rate-limiting enzymes in TCA cycle were up-regulated significantly at 36 h, as well as the genes encoding the succinyl-CoA synthetase (gismo_orf3725 and gismo_orf3726). And there were no genes showing obvious reduction in their mRNA levels in the TCA cycle. Three genes which involved in encoding pyruvate dehydrogenase complex (gismo_orf2295, gismo_orf4417 and gismo_orf3439) were induced significantly, indicating that the biosynthesis of acetyl-CoA from pyruvate might be promoted with high oxygen supply. The enhanced formation of acetyl-CoA was associated with the active TCA cycle. Meanwhile, the gene encoding PEP carboxykinase (gismo_orf3667), which played an important role in balancing the metabolite pool between the TCA cycle and glycolysis by catalyzing anaplerotic reaction from oxaloacetate (OAA) to PEP, was significantly up-regulated at 36 h. These up-regulated proteins and genes mentioned above suggested that relatively high activity of the TCA cycle provided sufficient ATP to maintain cell growth and promote cAMP production.

Promotion of 5-phosphoribosyl 1-pyrophosphate (PRPP) synthesis and purine metabolism is one of the reasons that why high oxygen supply enhanced cAMP production. Two PRPP synthesis related enzymes in PP pathway, ribulose-5-phosphate 3-epimerase and ribose-phosphate pyrophosphokinase and were considered to be overexpressed under high oxygen supply. Ribulose-5-phosphate 3-epimerase catalyzed the interconversion of ribulose-5-phosphate and xylulose-5-phosphate and its coding gene (gismo_orf2643) was obviously up-regulated at 36 h. Ribose-phosphate pyrophosphokinase is known to be implicated in the pyrophosphorylation of ribose-5-phosphate to PRPP as a regulatory enzyme in purine biosynthesis, and PRPP produced by this enzyme is of importance for purine biosynthetic pathway^21^. For instance, PRPP is one important effector molecule in the biosynthesis of inosine monophosphate (IMP) in *Bacillus subtilis*, and increasing cellular PRPP pool resulted in induction of gene expression in the purine biosynthetic pathway^22^. Here, we observed that the ribose-phosphate pyrophosphokinase (spot 211, gismo_orf1245) increased in its abundance with high oxygen supply, which was consistent with its mRNA change at 36 h. Higher expression levels of the two enzymes implied that more PRPP were supplied for purine biosynthesis, which benefited cAMP production. Additionally, several enzymes associated with purine metabolism were more abundant under high oxygen supply. Our previous studies showed that cAMP could be produced via de novo biosynthesis or salvage biosynthesis from additional hypoxanthine (HX) in this strain, and cAMP synthesis was mainly via the salvage route^23^. In the de novo route, phosphoribosylformylglycinamidine synthase (PFAS) catalyzes the fourth step of IMP synthesis. Our chip data displayed three genes (gismo_orf2828, gismo_orf2829 and gismo_orf2831) encoding PFAS II, PFAS I and purS respectively, were induced significantly with high DO level at least at one time point. In the salvage route, HX and PRPP are catalyzed to IMP by hypoxanthine phosphoribosyltransferase. Our study showed that hypoxanthine phosphoribosyltransferase (gismo_orf556) was up-regulated at the mRNA level at 36 h. Along with this, the adenylosuccinate synthetase (gismo_orf2817) which involved in adenine ribonucleotide biosynthesis showed positive change in its mRNA level at 24 h under high DO level. These data indicated that high oxygen supply might result in a more active purine biosynthesis and enhancement of carbon flux targeting to the desired product.

As shown in our microarray data, the genes involved in histidine metabolism, branched-chain amino acids (valine, leucine and isoleucine) metabolism and glutamate metabolism were mainly down-regulated under high oxygen supply. Two enzymes, phosphoribosyl-AMP cyclohydrolase (gismo_orf2631) and histidinol-phosphate phosphatase (gismo_orf998) which catalyzing the third and penultimate steps in histidine biosynthesis respectively, showed notable decreases in their mRNA levels at 24 h or 48 h. Meanwhile, the genes encoding urocanate hydratase (gismo_orf2746) and imidazolonepropionase (gismo_orf1167) which catalyzed the second and third steps of histidine degradation, respectively, were down-regulated significantly at 24 h or 48 h. Since purine nucleotides and the amino acid histidine both used PRPP as the biosynthetic precursors^24^, the remarkable decrease of four genes involved in histidine metabolism indicated more substrates were supplied for cAMP synthesis. The branched-chain amino acids are important for the synthesis of proteins and membranes probably representing cell growth, and their biosynthesis is regulated by some global transcriptional regulators of cellular metabolism, including carbon metabolism and nitrogen metabolism^25^. In most Gram-positive bacteria, the genes for the first two enzymes of the branched-chain amino acid biosynthesis (acetolactate synthase and ketol-acid reductoisomerase) belong to the same operon. In our study, the genes encoding acetolactate synthase (gismo_orf871 and gismo_orf872) and ketol-acid reductoisomerase (gismo_orf870) showed reductions at mRNA levels. Meanwhile, 2-isopropylmalate synthase (gismo_orf1668) and 3-isopropylmalate dehydrogenase (gismo_orf865) which participated in biosynthesis of L-leucine were both strongly repressed in their expression levels. Methylmalonate-semialdehyde dehydrogenase (gismo_orf2788) which related to branched-chain amino acids degradation was down-regulated as well. One gene encoding ABC branched-chain amino acid transporter (gismo_orf1905) was also found to be repressed significantly. Glutamate is one of the amino acids generated by the breakdown of the branched-chain amino acids. Eight genes involved in glutamate metabolism showed reduced expression under high oxygen supply, including L-glutamine synthetase (gismo_orf3527), glutamate synthase (NADH) small subunit (gismo_orf2622), glutamate synthase (NADH) large subunit (gismo_orf2623), L-glutaminase (gismo_orf3628), N-acetylglutamate synthase (gismo_orf518), gamma-glutamyltransferase (gismo_orf4371), and glutamate-cysteine ligase (gismo_orf1593 and gismo_orf2385). The lower activity of glutamine synthetase under high oxygen supply was probably due to the higher yield of cAMP, because expression of glutamine synthetase showed a decreased trend for glucose-grown cells when cAMP was present^26^.

Glutamine synthetase which catalyzes the condensation of glutamate and ammonia to form glutamine and glutamate synthase which catalyzes the synthesis of glutamate from 2-oxoglutarate and glutamine also play essential roles in nitrogen metabolism. Nitrogen is necessary for the production of amino acids, nucleotides, amino sugars, NAD, and so on. Ammonium is a preferential nitrogen source, as it can be assimilated directly into glutamine and glutamate^27^. And glutamine or glutamate act as sensed amino acids to indicate an internal nitrogen level^28^. Additionally, three genes encoding the enzymes which participate in nitrogen metabolism, including assimilatory nitrite reductase (NAD(P)H) (gismo_orf1222 and gismo_orf1223) and assimilatory nitrate reductase (NADH) (gismo_orf1215), were repressed significantly as well. In our study, high oxygen supply also displayed negative effect on ammonium transporter. Ammonium transport proteins are a class of integral membrane proteins that specifically shuttle ammonium across biological membranes. In bacteria, ammonium transport proteins are used to scavenge NH_4_^+^/NH3 from their environment for uptake and assimilation of nitrogen, and they facilitate ammonium uptake when the extracellular ammonium levels are low^29,30^. Four genes (gismo_orf826, gismo_orf1966, gismo_orf3118, gismo_orf3383) related to ammonium transport were found with significant down-regulated expression for at least two time points. The reduced activities of glutamate metabolism, nitrogen metabolism and ammonium transporter probably indicated nitrogen source was sufficient for cells under high oxygen supply.

In our study, high DO level results in an inevitable impact on some transcriptional regulators of *Arthrobacter* species, inducing AraC family and AsnC family transcriptional regulators and inhibiting PadR family and MarR family transcriptional regulators. The AraC family of bacterial transcriptional activators involves in diverse genetic systems, including regulation of the expression of some genes responded to stress and virulence such as antibiotics, organic solvents, and oxidative stress agents. For example, some members of the family control the production of virulence factors such as siderophores^31^. Comparative proteomics study also indicated that AraC transcriptional regulator may facilitate bacteria resistance to hexavalent chromium toxicity in *Shewanella oneidensis*^32^. AsnC is the first Lrp-related protein described in *E*. *coli*, and Lrp-related proteins are sometimes referred to as the Lrp/AsnC (leucine-responsive regulatory protein/asparagine synthase C products) family proteins which might be limited to bacteria and archaea. Lrp/AsnC family transcriptional regulators are known to regulate multiple cellular metabolisms globally (Lrp) or locally (AsnC)^33^. Members of the Lrp/AsnC family were reported to negatively regulate expression of branched-chain amino acid transport in *E*. *coli* or and *B*. *subtilis*^34,35^. For example, genes encoding a putative glutaminase and a putative amino acid/amine transport protein were negatively regulated responding to Lrp in *E*. *coli*. Therefore the repression of glutamate and branched-chain amino acid metabolism might be associated with induced AsnC family transcriptional regulator in our study. PadR proteins are involved in the regulation of the expression of enzymes participated in phenolic acid degradation and detoxification^36^. The PadR family is related to the bacterial and archaeal MarR family of transcriptional regulators which regulate a variety of biological functions, including resistance to multiple antibiotics and other toxic chemicals, adaptation to different environments and the expression of virulence factors^37^. In our study, two genes encoding PadR family transcriptional regulator (gismo_orf3382) and MarR family transcriptional regulator (gismo_orf886) both displayed significant down-regulation when cAMP achieved higher yield.

Some research suggested that the morphology of *Arthrobacter* cells was related to the cAMP levels of the cells, such as the cAMP levels in the rod stage cells are greater than in the spherical stage cells, or the extracellular cAMP levels increased significantly at the onset of stationary phase^38^. Biotin was reported to benefit the normal morphogenetic expression of *Arthrobacter* species^39^. The gene (gismo_orf4076) encoding biotin synthase which catalyzed the formation of biotin was up-regulated under high oxygen supply, suggesting that higher yields of cAMP might be related to normal morphogenesis in this strain. The control of cAMP levels was considered to associate with cAMP excretion, cAMP hydrolysis by phosphodiesterase, and cAMP formation by adenylate cyclase (AC). Since AC and phosphodiesterase exhibited nonsignificant changes under high oxygen supply, we hypothesized that massive accumulation of extracellular cAMP was due to efficient cAMP export in our strain. The mechanism of cAMP exit has been ascribed to an energy-dependent extrusion pump. For example, MRP4, -5, and -8, which were members of the multidrug-related protein (MRP) family, had been shown to their ability to transport cAMP^40^. One gene, *abcB3*, which was one of the ATP-binding cassette (ABC) transporters, could export cAMP in *Dictyostelium discoideum*^41^. AC toxin is known as a novel protein toxin, which is discovered by the presence of AC catalytic activity in commercial pertussis vaccines. The overproducing cAMP from this toxin is able to disorganize cellular signaling processes and cause cell death^42^. Thus we supposed that the exporter of cAMP might belong to a kind of drug efflux transporters. The microarray results showed that one gene (gismo_orf371) encoding Major Facilitator Superfamily transporter was induced obviously. However, the mechanism of cAMP secretion in *Arthrobacter* species remains poorly elucidated and the functions of multidrug transporters and ABC transporters of *Arthrobacter* species were largely unknown. Therefore further investigations need to be done in order to test the hypothesis and to explain the cAMP export system in this strain.

The expression profiles of proteins and mRNAs are hypothesized to be strongly correlated based on the central dogma of molecular biology. However, the correlation between mRNA and protein abundances has been reported to be poor in most reports^43^. The discrepancy between mRNA and protein levels may be due to stabilities of RNA and protein, functional categories of a given gene/protein, protein regulation by post-translational modification, post-transcriptional regulation of protein amounts, experimental measurement errors, deficiency of mathematical analysis, and so on^44–46^. Some researches exhibited that expression levels of proteins and mRNAs correspond reasonably well on the whole, except some proteins and genes showing discordance^47^. Several studies showed that only 20–40% of the changes in protein levels were attributed to variable mRNA levels^48^. Moreover, many studies showed only a weak correlation or no correlation between mRNA and protein abundances^49^. In our study, 25 out of 54 successfully identified proteins exhibited the expression pattern disagreement between their abundances and mRNA levels. For instance, the protein abundance of 6-phosphogluconate dehydrogenase was down-regulated at 36 h, while the corresponding genes were up-regulated (Table 1 and Supplementary Table S1). And only 11 identified proteins showed quantitative agreement with their corresponding genes (a similar fold change in expression levels). However, this variation illustrates the importance of combination of the transcriptomics and proteomics data analysis. The transcriptomics and proteomics data can be used to complement each other in order to avoid analytic variations from each technology, or the detection of a good mRNA-protein correlation can improve the credibility of data analysis^50^.

There are also some limitations in our microarray and 2D results. Due to insufficiency of the *Arthrobacter* species gene databases, we still have a lot of genes which cannot match any known genes. And the method of 2D gel electrophoresis has many limitations, such as solubility problems and difficulties of detection of low abundance proteins^51^. In our proteomic analysis, 13 out of 67 spots failed in protein identification. Thus further verification and functional analysis are needed to confirm the results of our omics data analysis and to elucidate the molecular mechanism of cAMP production.

## Methods

### Microorganism, media and culture conditions

The strain used for biosynthesis of cAMP was *Arthrobacter* sp. CGMCC 3584 that isolated and stored in our laboratory. The seed culture medium contained in grams per liter: glucose 10, peptone 10, beef extract 10, and NaCl 3. The fermentation medium contained in grams per liter: glucose 50, urea 8, K_2_HPO_4_ 18, KH_2_PO_4_ 5, MgSO_4_ 0.1, biotin 0.01, hypoxanthine 8, NaF 0.4 and CoCl_2_ 0.005. The initial pH was adjusted to 7.0 by NaOH and the medium was autoclaved for 20 min at 115 °C. The seed cultures were incubated at 30 °C and 300 rpm for 18 h. Then the seed medium was transferred into a 5-L fermenter (NBS Bioflo-110) containing 3 L of fermentation medium at an inoculum dose of 10% (v/v). The fermentation parameters were controlled by a digital measurement and control system. The cultivation was carried out at 30 °C and aeration rate was 8 L/min. The pH was automatically controlled to within 7.0–7.2 with 2.0 M NaOH and 2.0 M HCl. Peanut oil (0.1%, v/v) added as an antifoaming agent. The agitations were set at 150 r/min and 350 r/min to construct low oxygen supply and high oxygen supply conditions, respectively. Tests were carried out in triplicates and the mean values were calculated for fermentation parameters.

### Sample collection

Samples (three biological replicates) were respectively collected at 12 h, 24 h, 36 h and 48 h under two different oxygen supply conditions for transcriptional analysis. Equal samples of each of the independent biological replicates were mixed together for DNA microarray analysis. For 2D gel separation, samples (three biological replicates) were respectively collected at 12 h and 36 h under two different oxygen supply conditions. The cells were pelleted by centrifugation at 5000 × *g* for 10 min at 4 °C and rinsed twice with ice-cold PBS buffer (137 mM NaCl, 2.7 mM KCl, 8 mM Na_2_HPO_4_, and 2 mMKH_2_PO_4_, pH 7.40). All the cells were frozen immediately using liquid nitrogen and then stored at −80 °C.

### Genome Sequence

Whole-genome sequencing of *Arthrobacter* sp. CGMCC 3584 was performed using the Roche 454 Genome Sequencer FLX System (Majorbio, Shanghai, China). After the removal of duplications (by cd-hit-454), the clean data were assembled using GS Assembler. The finished results were analyzed and annotated using Glimmer, GeneMarker, Gismo, epos-blastview, blast2go, tRNAscan-SE, rnammer. The gene function annotation was predicted by using the Kyoto Encyclopedia of Genes and Genomes (KEGG), Clusters of Orthologous Groups (COG), and Gene Ontology (GO). Finally, 4,075 coding sequences (CDSs) were predicted and based on this information the following experiments were carried out.

### Transcriptome analysis

The *Arthrobacter* microarrays were customized using Agilent eArray program according to the manufacturer’s recommendations. Each customized microarray (8 × 15 K) contained spots with 4,075 gene-specific 60-mer oligonucleotides representing the 4,075 protein-coding genes in *Arthrobacter* sp. CGMCC 3584. Total RNA was amplified and labeled by Low Input Quick Amp Labeling Kit, One-Color (Cat#5190-2305, Agilent technologies), following the manufacturer’s instructions. Labeled cRNA were purified by RNeasy mini kit (Cat#74106, QIAGEN). Each Slide was hybridized with 600ng Cy3-labeled cRNA using Gene Expression Hybridization Kit (Cat#5188-5242, Agilent technologies) in Hybridization Oven (Cat#G2545A, Agilent technologies), according to the manufacturer’s instructions. After 17 hours hybridization, slides were washed in staining dishes (Cat#121, Thermo Shandon) with Gene Expression Wash Buffer Kit (Cat#5188-5327, Agilent technologies), followed the manufacturer’s instructions. Slides were scanned by Agilent Microarray Scanner (Cat#G2565CA, Agilent technologies) with default settings, Dye channel: Green, Scan resolution 5μm, PMT 100%, 10%, 16 bit. Data were extracted with Feature Extraction software 10.7 (Agilent technologies,). Raw data were normalized by Quantile algorithm, Gene Spring Software 11.0 (Agilent technologies). Genes with fold change bigger than 2 were considered to be significantly differential expression genes responding to different oxygen supply.

### qRT-PCR

Five selected genes were further validated by qRT-PCR. Gene-specific primers used for qRT-PCR were listed in Supplementary Table S3. QRT-PCR was carried out by the 7900 HT Sequence Detection System (ABI, USA) using ABI Power SYBR Green PCR Master Mix (ABI, USA) according to the manufacturer’s instructions. The fold change of each transcript in each sample relative to the control sample was measured in triplicates. The 16 S rRNA gene was considered as an endogenous reference. And the calculation of differential expression level was based on comparative threshold cycle method^52^.

### Proteome analysis

The samples were grinded to power with liquid nitrogen and then dissolved in lysis solution at 30 °C for 1 hour. The solution was centrifuged by 15000 *g* for 15 min at room temperature. The clear supernatant was collected and stored at −80 °C for isoelectric focusing (IEF). The protein concentration was determined by the bradford method. IEF was carried out on an IPGhor IEF System (GE Healthcare) with 300 μg of protein (450 μl) at 20 °C using the 24 cm drystrips (GE Healthcare, pH = 4–7). After equilibration, SDS-PAGE electrophoresis was performed by Ettan-DALT-Six system (GE Healthcare) at 15 °C. The gel was run for 45 minutes at 100 V then for 6–8 hours at 200 V. The gel was visualized by silver stain as described by Shevchenko *et al*.^53^. The stained gel was then scanned by the Image Scanner (GE Healthcare, USA) at a resolution of 300 dots per inch. All gel images were analyzed using PDquest 8.0 software. A threshold of p ≤ 0.05 and fold change ≥2.5 or ≤0.4 was used to select differentially protein spots. *P*-value was performed with Students *t* test. Selected protein spots were excised from gels and in-gel digestion with trypsin was performed as described previously^54^. Peptide MS and MS/MS were performed on an Ultraflex TOF/TOF mass spectrometer (Bruker). Both the MS and MS/MS data were integrated by Mascot2.3 (Matrix Science) for protein identification.

### Data availability

CDSs description:

1. Gene Expression Omnibus (GEO) accession GPL19020.

2. https://www.ncbi.nlm.nih.gov/geo/query/acc.cgi?acc=GPL19020.

Gene expression data:

1. Gene Expression Omnibus (GEO) accession GSE99546.

2. http://www.ncbi.nlm.nih.gov/geo/query/acc.cgi?acc=GSE99546.

KEGG pathway database^55^:

1. Map00010, 00020, 00030, 00230, 00250, 00280, 00290, 00340, 02060.

2. http://www.genome.jp/kegg/pathway.html.

## Electronic supplementary material

Supplementary Information

Table S1

Table S2
