# Supplementary material for: Comparative transcriptomic and proteomic analysis of Arthrobacter sp. CGMCC 3584 responding to dissolved oxygen for cAMP production
Source: Sci Rep. 2018 Jan 19;8:1246. doi: 10.1038/s41598-017-18889-4 (PMC5775200; doi:10.1038/s41598-017-18889-4)
Supplement: Supplementary file 1 — Supplementary Information [file 41598_2017_18889_MOESM1_ESM.doc]

**Supplementary Information**

**Comparative** **transcriptomic and proteomic analysis of *Arthrobacter* sp. CGMCC 3584 responding to dissolved oxygen for cAMP production**

Huanqing Niu, Junzhi Wang, Wei Zhuang, Dong Liu, Yong Chen, Chenjie Zhu & Hanjie Ying^*^

**Table S3 Primers used for qRT-PCR**

| Seq. Description | Primer Name | Sequence (5'to3') |
| --- | --- | --- |
| pyruvate kinase | CUST_5804_PI428272168 Forward Primer | CGCAAAAATTGTGGCTACGTT |
|  | CUST_5804_PI428272168 Reverse Primer | TCCAGTACCGCGAGTGTGTTT |
| fructose-bisphosphate aldolase | CUST_5998_PI428272168Forward Primer | CAAGAACTACGGCGTCAACATC |
|  | CUST_5998_PI428272168Reverse Primer | GGACAAAGCCGTCCAGCTT |
| phosphoglycerate kinase | CUST_7039_PI428272168Forward Primer | CAAACACCCTCAACGAACTCATC |
|  | CUST_7039_PI428272168Reverse Primer | CAGGTCACTTCGAACCAGAATG |
| succinyl-CoA synthetase subunit beta | CUST_6873_PI428272168 Forward Primer | TGAGCACTCCACCAACATCCT |
|  | CUST_6873_PI428272168 Reverse Primer | CTGGGCAATCATCACCTTGTTA |
| ribose-phosphate pyrophosphokinase | CUST_4456_PI428272168 Forward Primer | GCTGATGGAACAGCTGATCATG |
|  | CUST_4456_PI428272168 Reverse Primer | TGGCGGGCGTAGGGATA |
| 16S rRNA | 16s Forward Primer | ATGCGTAGCCGACCTGAGA |
|  | 16s Reverse Primer | GCTGCCTCCCGTAGGAGTCT |
